# Shaping the Future of Coffee: Climate Resilience, Liberica’s Rise, and By-Product Innovation—Highlights from the International Coffee Convention 2023 (ICC2023)

**DOI:** 10.3390/foods13060832

**Published:** 2024-03-08

**Authors:** Dirk W. Lachenmeier, Philipp Weller, Adriana Farah, Ma. Carmen Ablan Lagman, Massimiliano Fabian, Maria Dolores del Castillo, Steffen Schwarz

**Affiliations:** 1 Chemisches und Veterinäruntersuchungsamt (CVUA) Karlsruhe, Weissenburger Strasse 3, 76187 Karlsruhe, Germany; 2 Institute for Instrumental Analytics, Faculty of Biotechnology, Mannheim University of Applied Sciences, 68163 Mannheim, Germany; p.weller@hs-mannheim.de; 3 Nutrition Institute, Federal University of Rio de Janeiro, Rio de Janeiro 21941-902, Brazil; afarah@nutricao.ufrj.br; 4 Practical Genomics Laboratory, Center for Natural Science and Environment Research, De La Salle University, Manila 1004, Philippines; ma.carmen.lagman@dlsu.edu.ph; 5 Demus S.p.A., via Caboto, 31, 34147 Trieste, Italy; mfabian@demus.it; 6 Instituto de Investigaciónen Ciencias de la Alimentación (CIAL), Consejo Superior de Investigaciones Científicas-Universidad Autónoma de Madrid (CSIC-UAM), 28049 Madrid, Spain; mdolores.delcastillo@csic.es; 7 Coffee Consulate, Hans-Thoma-Strasse 20, 68163 Mannheim, Germany; schwarz@coffee-consulate.com

**Keywords:** coffee sustainability, *Coffea liberica*, climate resilience, coffee by-products, health benefits, traceability, authentication, coffee consumption, regulatory frameworks, innovation in coffee industry

## Abstract

The International Coffee Convention 2023 comprehensively addressed the contemporary challenges and advancements in the coffee industry, emphasizing sustainability, health, and innovation. This convention gathered experts and stakeholders to explore diverse aspects of coffee, ranging from the potential of underutilized species like *Coffea liberica* in terms of climate resilience to the innovative use of coffee by-products. The convention featured presentations and discussions, employing both empirical research and analytical reviews to explore various topics, including the health benefits of coffee, the advancements in traceability and authentication methods, and the impact of global regulatory changes on coffee production and trade. The key findings highlighted the importance of biodiversity in coffee production as a response to climate change, the significant health benefits and sustainability potential of coffee by-products, and the evolving landscape of coffee consumption patterns driven by technological innovations. The convention also stressed the need for alignment in global coffee trade regulations, particularly concerning deforestation and traceability. The 2023 convention underscored the complexity and interconnectivity of the coffee industry’s challenges and opportunities. It concluded with a forward-looking perspective, emphasizing the need for continued research, sustainable practices, and collaborative efforts to shape the future of the coffee industry. The community is looking forward to furthering these discussions at the next International Coffee Convention in 2024.

## 1. Introduction

Following a series of international meetings organized by Coffee Consulate in Mannheim, namely the Stuttgart Coffee Symposium 2018, Stuttgart Coffee Symposium 2020, and the Virtual Coffee Symposium 2021, which was an online event during the COVID-19 pandemic [1], and also a topical live session of The 2nd International Electronic Conference on Foods—“Future Foods and Food Technologies for a Sustainable World”—entitled “Coffee By-products as Sustainable Novel Foods” [2], the International Coffee Convention 2023 (ICC2023) is continuing this tradition in a renewed fashion with an expanded scope. Convening at the Congress Center Rosengarten, Mannheim, Germany, from 30 September to 3 October, the ICC2023 marked a significant advancement in the discourse surrounding coffee science and the coffee industry. The ICC2023 conference showcased a series of keynote speeches from experts, along with a range of presentations covering diverse subjects from broad, interdisciplinary angles [3]. The primary areas of focus encompassed the sustainability and innovative use of coffee by-products and the role of biodiversity in coffee cultivation amidst climate change challenges, with particular attention given to *Coffea liberica*. Additional topics discussed were the health implications of coffee consumption, evolving consumer preferences in coffee drinking, emerging technologies shaping the future of coffee, and the industry’s current challenges and prospects. The event also highlighted the importance of traceability and authenticity in coffee production. The conference culminated with the prestigious Kaldi Award ceremony, recognizing outstanding contributions to the coffee sector.

This report, compiled by the scientific advisory board members of the conference, synthesizes the key discussions and outcomes of the ICC2023. It offers a comprehensive overview of the 32 presentations at the convention, which attracted participation from over 150 people, facilitating a dynamic exchange of ideas. The sessions, accessible to a wider audience through video recordings, underscored the evolving scientific understanding and practical applications of coffee by-products, tracing their trajectory from initial research to modern sustainable applications.

**Figure 1:**
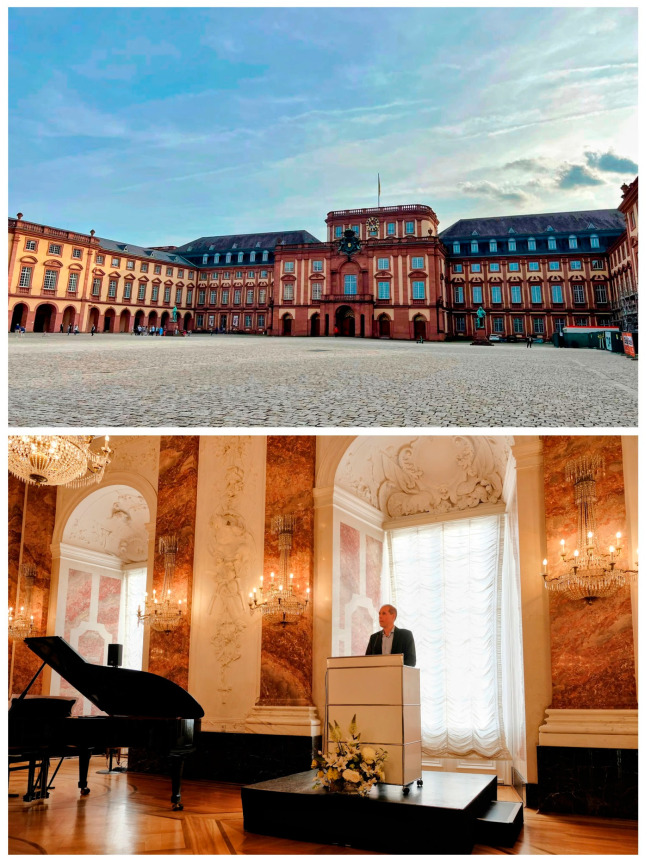
Opening of the ICC2023 conference at the historic Rittersaal (Knights’ Hall) of the baroque Mannheim Palace ((**upper panel**): the baroque Mannheim Palace, outside view; (**lower panel**): Steffen Schwarz at his congress opening speech). Permission has been obtained from all individuals in the image.

The opening of the ICC2023 conference was held in the historic Rittersaal (Knights’ Hall) of the baroque Mannheim Palace, a venue steeped in cultural and architectural significance (Figure 1). This choice of location mirrored the conference’s theme of exploring the rich history and future potential of coffee.

**Figure 2:**
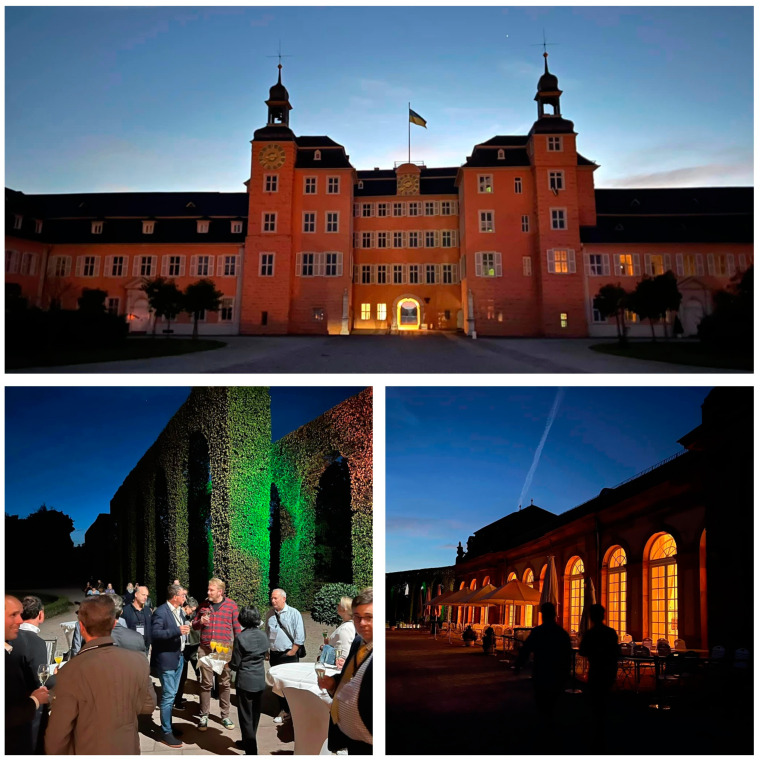
Social event at Schwetzingen Castle. Permission has been obtained from all individuals in the image.

A notable event in the conference’s social program was hosted at Schwetzingen Castle, Schwetzingen, Germany, particularly significant for its historical connection to the emerging of coffee culture in Europe (Figure 2). It was under the auspices of Kurfürst Carl Theodor (reigning prince-elector of the Palatinate), in the 18th century, that Schwetzingen Castle’s Orangerie became a venue for the cultivation of exotic plants, including coffee. The cultivation of coffee was conducted on a scale sufficient to meet the needs of the Kurfürst’s household, and the roasting of these beans was meticulously carried out in the castle kitchen [4]. This early adoption and cultivation of coffee plants within the castle grounds exemplified a period of agricultural and botanical experimentation that preluded coffee’s widespread popularity in Europe.

The event at Schwetzingen Castle provided attendees with a direct link to this historical period, underscoring the long-standing relationship between coffee and European culture. The castle’s historical association with the early cultivation of coffee in the region was a poignant reminder of the journey of coffee from an exotic curiosity to an integral part of social and economic life in Europe. This combination of historical setting and contemporary scholarly discourse offered a unique perspective for the ICC2023 conference, aligning with its objectives to explore both the historical context and evolving dynamics of coffee in a global setting.

## 2. Coffee Sustainability and By-Products

This was a major topic of the conference, with eight presentations centered around a comprehensive exploration into the innovative uses and economic potentials of coffee by-products, delving into their multifaceted roles in sustainability and health promotion, as well as industry challenges.

Rieke-Zapp [5] emphasized the importance of a fruit-centered coffee process for optimal financial benefits and sustainability. He argued that focusing on the seed alone does not denote excellent cherry quality nor provide the maximal financial benefit to farmers. In a similar fashion, Iriondo-DeHond and del Castillo [6] highlighted the newly authorized traditional food status of dried coffee cherries, commonly known as cascara, in the European Union. They emphasized cascara’s richness in nutrients and bioactive compounds that are beneficial for health, proposing its use in various beverage forms. Their research focused on cascara’s valorization, aiming to enhance the coffee industry’s sustainability and contribute to achieving the Sustainable Development Goals. Cantergiani [7] also explored the volatile fraction of cascara from various origins and processes. His study characterized these components to understand their sensorial properties, and he proposed a cascara flavor wheel based on this sensory evaluation, highlighting cascara as a potential ingredient in the food and beverage sector.

Kull et al. [8] discussed the regulatory aspects and correct labeling of coffee by-products in the EU market. They shed light on the challenges posed by novel food regulations, particularly for traditional foods from third countries, and the strict approval requirements hindering the practical utilization of coffee by-products. Similarly, Lachenmeier and Walch [9] critiqued the EU’s novel food regulation, arguing that it poses a significant trade barrier to coffee by-products. They advocated for a revision of the regulation to allow for a rapid and flexible introduction of novel foods, including traditional foods from third countries, to uphold food security and sustainability in the EU.

Rigling et al. [10] presented their research on underutilized coffee leaves and flowers. They analyzed these by-products for their sensory characteristics and aroma profiles, finding a wide range of flavors and identifying key volatile compounds. This research opens up their potential applications in the food market and contributes to making coffee growing more sustainable.

Rennert [11] investigated the use of coffee by-products as antioxidants to stabilize bioplastics. His study showed that the compounds like polyphenols and vitamins in coffee by-products could prevent oxidative processes and ageing in bioplastics, offering a recyclable potential for the materials and bio-based plastics. Rennert’s findings highlighted the differences in coffee by-products’ extrudability, mechanical properties, and successful thermo-oxidative stabilization when compounded with bio-based polymers.

Lastly, Kowa [12] presented on the economic and ecological potentials of waste biomass generated from coffee planting and harvesting. Kowa’s discussion unveiled the possibilities for using these materials to achieve climate targets, soil improvement, CO_2_ reduction, and economic benefits for farmers.

**Figure 3:**
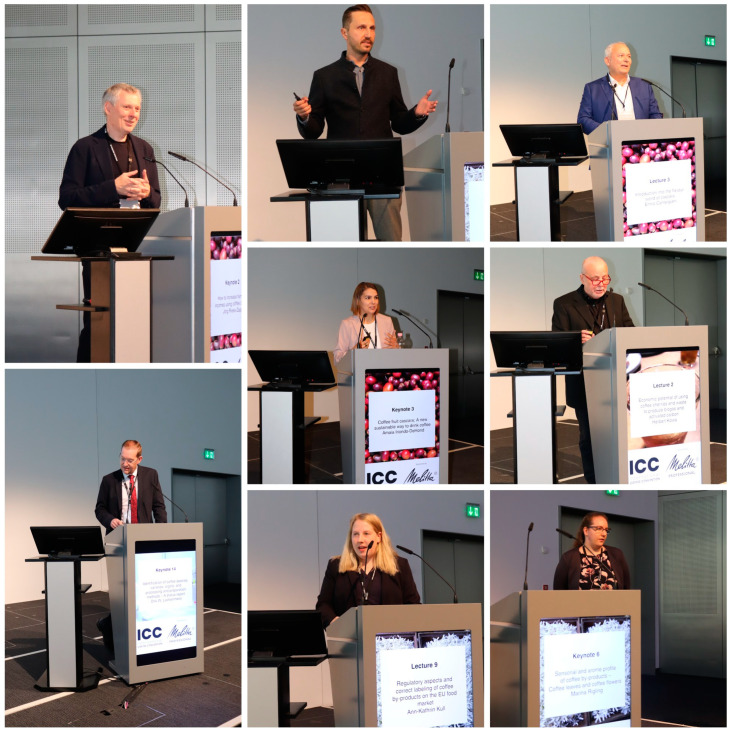
Speakers on the topic of coffee sustainability and by-products (from left to right; (**upper panels**): Rieke-Zapp, Rennert, Catergiani; (**middle panels**): Iriondo-DeHond, Kowa; (**lower panels**): Lachenmeier, Kull, Rigling). Permission has been obtained from all individuals in the image.

These presentations (Figure 3) collectively emphasized the importance of sustainable practices, innovative applications, and regulatory adaptations in maximizing the potential of coffee by-products, aligning with the goals of environmental sustainability and economic viability in the coffee industry.

## 3. *Coffea liberica*, Climate Resilience, and Biodiversity

Another main topic of the conference, comprising six presentations, covered various aspects of *Coffea liberica*, its role in climate resilience, and the broader theme of biodiversity in coffee cultivation.

Wee Ting Lee [13] examined the past, present, and future potential of the Liberica coffee industry in Malaysia. He focused on the challenges that the industry faces amidst the specialty coffee movement and global warming. His work highlighted the history of Liberica coffee cultivation among indigenous communities in Borneo, emphasizing the diversity of Liberica varieties and their potential, from rainforest highlands to river valleys. The “Liberica refinement project” in Sarawak, aiming to empower indigenous communities and improve their livelihoods through market-driven approaches, was a key focus.

Kwok [14] presented on the sensory attributes of Liberica coffee with different origins, processing methods, elevations, and roasting techniques. Kwok’s work contributed significantly to understanding the unique sensory properties of Liberica coffee, a species that has recently re-emerged from the shadow of Arabica and Canephora.

Kiefer et al. [15] explored the aromatic fingerprint of fermented *Coffea liberica*. Their study investigated the impact of fermentation on the sensory profile of green and roasted coffee beans, using gas chromatography–mass spectrometry/ion mobility spectrometry (GC-MS/IMS) to analyze the volatile compounds of fermented and unfermented Liberica coffee.

Ablan Lagman [16] presented her work on the variability in Philippine *Coffea liberica*. She explored the potential of *C. liberica* and *C. liberica var. dewevrei*, which are produced widely in the Philippines, in terms of their unique flavor profiles and potential to thrive in a warming climate. Her study included evaluating the bean morphology, chlorogenic acid and caffeine content, and genetic variability of *C. liberica* varieties, providing insights into areas of increased production in the Philippines.

Schäfer [17] discussed the “International Conservation Collection of Coffee Varieties” at Wilhelma, Stuttgart, Germany. This initiative represents a significant step towards preserving the diversity of coffee cultivars, reflecting the growing recognition of the importance of biodiversity in coffee cultivation.

Finally, Montagnon’s presentation [18] challenged the conventional approach to agronomic research in coffee farming. He advocated for a refreshed approach focusing on coffee farmers’ profitability rather than just yield, emphasizing the importance of understanding farmers’ limitations and developing practices that address these constraints.

**Figure 4:**
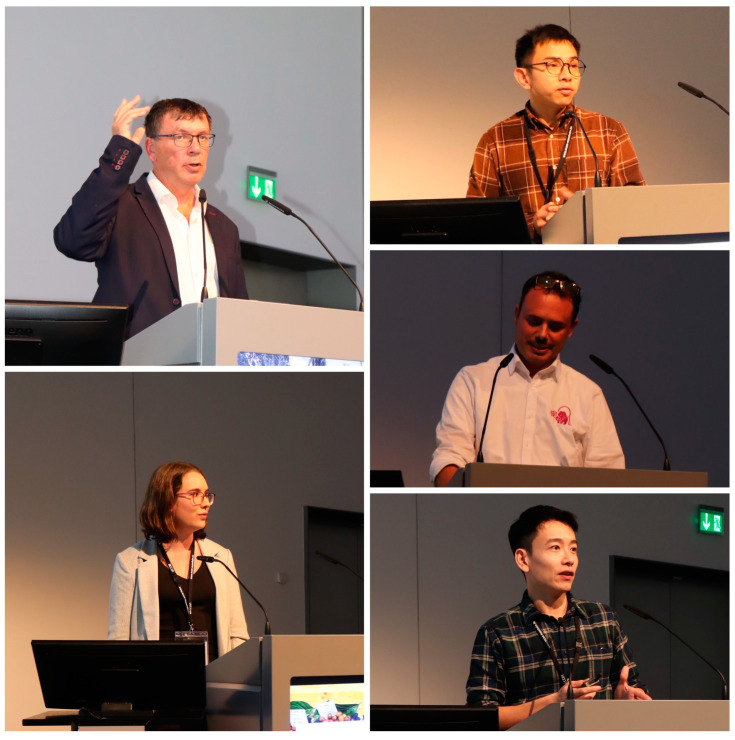
Speakers on the topic of *Coffea liberica*, climate resilience, and biodiversity (from left to right; (**upper panels**): Montagnon, Wee Ting Lee; (**middle panel**): Schäfer; (**lower panels**): Kiefer, Kwok). Permission has been obtained from all individuals in the image.

Overall, the significance of *Coffea liberica* in the context of climate change and biodiversity was highlighted. These presentations provided valuable insights into the unique characteristics of Liberica coffee, its potential in the specialty coffee market, and the importance of sustainable and profitable farming practices (Figure 4). This session underscored the need for continued research and development to enhance the sustainability of coffee cultivation and the resilience of coffee varieties in the face of changing environmental conditions.

## 4. Innovations in the Coffee Business, Technology, and Consumption Patterns

The next topic, comprising six presentations, delved into the evolving landscape of the coffee industry, exploring the intersection of innovation, technology, and shifting consumer behaviors.

Peluso’s presentation [19] highlighted the transformative potential of coffee by-products in the industry (see also Section 2). Peluso emphasized the economic value derived from utilizing these by-products in various applications, from energy production to the development of functional food ingredients and nutraceuticals. His insights shed light on the role of coffee by-products in driving sustainability and innovation, marking a shift from waste to wealth.

Kohler [20] explored the application of Design Thinking in the coffee industry. She detailed how this human-centered approach can identify new business opportunities and address complex challenges, using examples from projects conducted at the Mannheim University of Applied Sciences. Kohler’s insights into Design Thinking provided a fresh perspective on innovation pathways in the coffee sector.

Zimmermann [21] discussed Business Model Innovation (BMI) as a strategy for the small- and medium-sized enterprises (SMEs) in the coffee sector facing various challenges. Zimmermann underscored the importance of sustainability-oriented BMI and its potential to offer solutions to the multifaceted issues faced by the industry.

Müller [22] presented on digitalization in coffee roasting. He emphasized the potential for innovation through digital ecosystems that enhance user experiences of coffee roasting. Müller’s presentation showcased the importance of technological platforms in fostering collaboration and value creation in the craft of coffee roasting.

Peluso [23] also addressed the challenges and adaptation strategies in the changing coffee business landscape. He explored how climate change, consumer demand for sustainable practices, and the COVID-19 pandemic have reshaped the industry, highlighting the need for businesses to be agile and embrace digital transformation.

Kaschefi’s contribution [24] focused on the importance of coffee and food solutions in modern workplaces. He discussed how coffee breaks and the availability of quality coffee options enhance cognitive function, productivity, and social interactions in the workplace. Kaschefi also highlighted the evolving requirements of modern vending concepts in offices and the catering sector.

**Figure 5:**
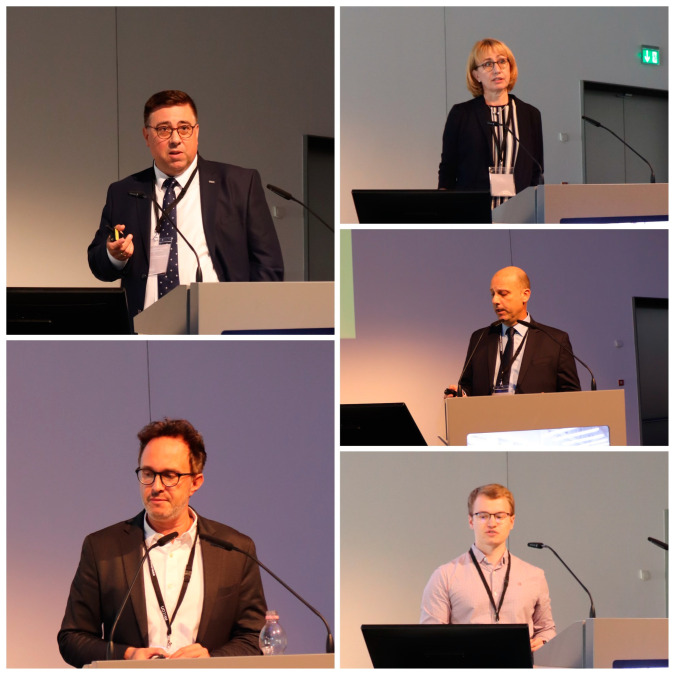
Speakers on the topic of innovations in the coffee business, technology, and consumption patterns (from left to right; (**upper panels**): Zimmermann, Kohler; (**middle panel**): Peluso; (**lower panels**): Kaschefi, Müller). Permission has been obtained from all individuals in the image.

This conference topic provided a comprehensive overview of the current trends and future directions in the coffee industry (Figure 5). The presentations collectively underscored the need for embracing innovative approaches, leveraging digital technologies, and responding to changing consumer preferences to navigate the dynamic coffee business landscape successfully.

## 5. Coffee and Health

The “Coffee and Health” topic of the conference, comprising five presentations, offered an exploration of the intersection between coffee consumption and health outcomes. This session included a range of studies shedding light on the potential health benefits of coffee and its by-products.

Farah [25] opened the discussion with a presentation of the role of coffee by-products in the promotion of sustainable health. Farah highlighted the significant contributions of these by-products to a sustainable food system, emphasizing their nutritional and bioactive properties and the role of fermentation in their contribution to health. The talk underlined the need for innovative approaches to harness the health benefits of coffee by-products while promoting sustainability in the coffee industry.

Machado et al. [26] presented research on the colonic fermentation of coffee melanoidins and their potential cardioprotective effects. Their study demonstrated how coffee melanoidins might influence cholesterol metabolism, suggesting a hypocholesterolemic effect that contributes to cardiovascular health.

Del Castillo and Iriondo-DeHond [27] discussed food security opportunities from plant to coffee cup. They emphasized the importance of transforming the coffee industry to combat food insecurity and malnutrition, advocating for the valorization of coffee by-products. Their presentation highlighted the potential of these by-products to contribute significantly to global food security and nutrition goals.

Sánchez-Martín et al. [28] explored the health benefits of Instant Cascara, a beverage derived from dried coffee cherries. Their research focused on the potential of Instant Cascara to promote gastrointestinal health and prevent colon cancer, showcasing its impact on key physiological cell events.

Finally, Rawel and Sagu [29] presented on the potential of green coffee proteins as new functional food components. Their study delved into the roles of the proteins, enzymes, peptides, and free amino acids in green coffee beans in flavor development and the formation of coffee’s aroma. Their research highlighted the unique properties and potential applications of these proteins in the food, cosmetic, and pharmaceutical industries.

**Figure 6:**
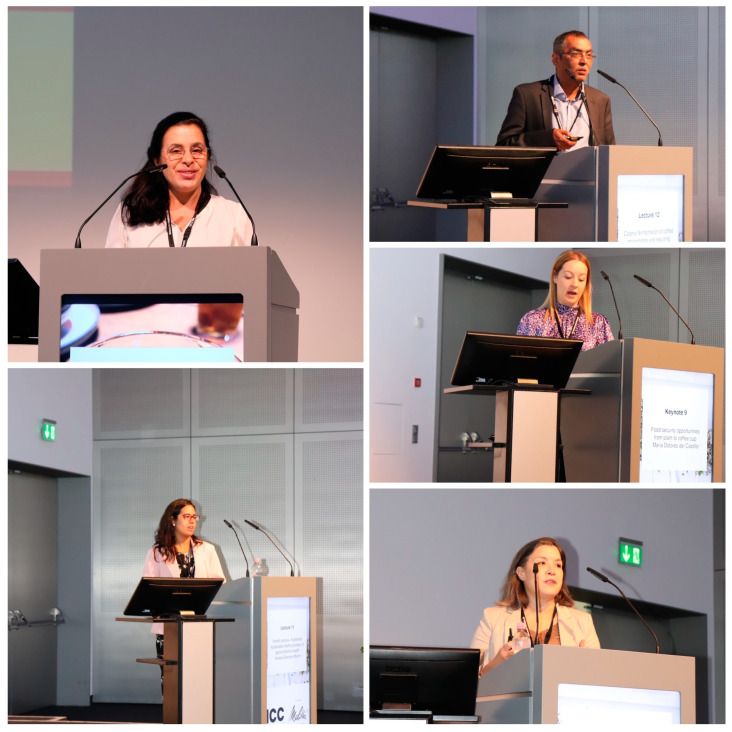
Speakers on the topic of coffee and health (from left to right; (**upper panels**): Farah, Rawel; (**middle panel**): Sánchez-Martín; (**lower panels**): Machado, del Castillo). Permission has been obtained from all individuals in the image.

The presentations on “Coffee and Health” offered insightful perspectives on the health-promoting properties of coffee and its by-products (Figure 6). The presentations collectively underscored the importance of further research and innovation in this field, contributing to the development of sustainable and health-promoting coffee products.

## 6. Coffee’s Traceability, Authentication, and Future Challenges

The last topic explored various facets of the coffee industry, focusing on challenges and innovations. The presentations in this section provided insights into diverse topics ranging from global market challenges, advancements in analytical methods for coffee authentication, and the implications of international agreements on the coffee community, to the evolution of traceability systems in the coffee sector.

Fabian’s initial presentation [30] highlighted the multifaceted challenges facing the global coffee market in 2023, including regulatory changes in the European Union regarding glyphosate usage and the European Parliament’s position on the Directive on Corporate Sustainability Due Diligence (CSDDD). He emphasized the need for a readiness to fulfill new EU legislations, especially concerning smallholder coffee farming families. A key focus was the European Union’s deforestation regulation, aimed at minimizing the EU market’s contribution to deforestation and forest degradation. This regulation underscores the urgent need for guidelines and strategies to differentiate between forest and coffee agroforestry systems, ensuring sustainable coffee farming practices that align with global efforts to combat climate change and protect biodiversity.

Montagnon’s presentation (later published in an expanded format by Lachenmeier and Montagnon [31]) discussed the implications for and compliance strategies of the global coffee community concerning the International Treaty on Plant Genetic Resources for Food and Agriculture (ITPGRFA) and the Nagoya Protocol on Access and Benefit-sharing of the Convention on Biological Diversity (CBD). His presentation underscored the importance of these agreements in conserving coffee’s genetic resources and ensuring fair benefit distribution, while also acknowledging the complexities and implementation challenges that they present.

Lachenmeier [32] provided an overview of the current state of the art of coffee identification techniques, setting the stage for subsequent specialized talks in the session. Lachenmeier’s contribution underscored the importance of advanced analytical methods in maintaining coffee quality and integrity, fostering sustainable practices in the industry and driving innovation in coffee product development.

In this area, Fabian’s second presentation [33] introduced an innovative service model that integrates genetic, chemical, and sensory analyses of coffee, linking them to a QR code. This approach aims to enhance transparency for both buyers and sellers, from bean to cup, ensuring compliance with the declared quality.

Teipel [34] explored the use of Nuclear Magnetic Resonance spectrometry (NMR) in coffee authentication. His work demonstrates how NMR, combined with a targeted quantitative analysis and non-targeted multivariate analysis, can verify product claims like geographical origin, botanical variety, and farming method. Teipel’s research highlights the precision and potential of NMR in addressing food fraud in the coffee industry.

Wintel et al. [35] provided insights into the use of isotopic fingerprinting as an alternative tool for coffee authenticity checks. Their research highlighted how stable isotope analysis could disclose food authenticity and apply quality checks to coffee, demonstrating the technique’s potential in determining geographical origin and growing conditions.

Weller et al. [36] delved into the role of modern untargeted benchtop analytical strategies in coffee research. They discussed the principles and examples of benchtop “volatilomics” approaches in food and fermentation processes, showcasing how these techniques could be utilized directly where needed in the future.

**Figure 7:**
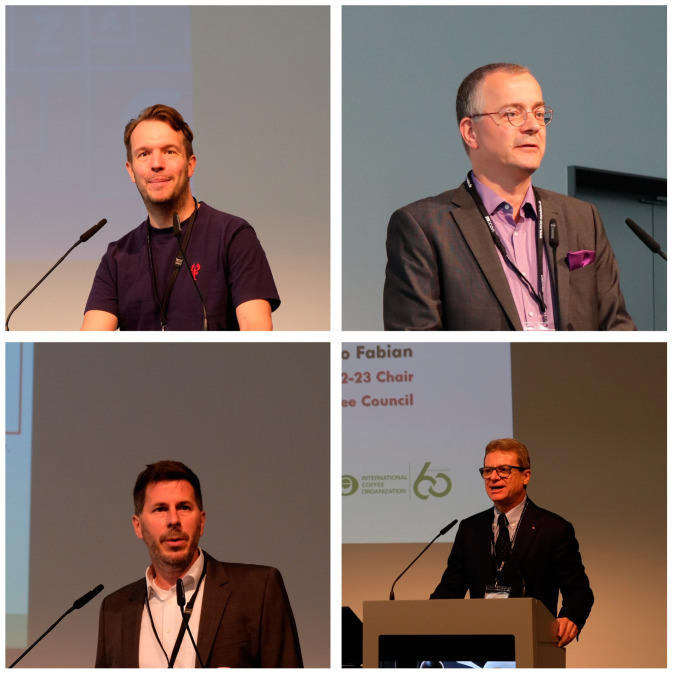
Speakers on the topic of coffee traceability, authentication, and future challenges (from left to right; (**upper panels**): Wintel, Teipel; (**lower panels**): Weller, Fabian). Permission has been obtained from all individuals in the image.

Collectively, these presentations emphasized the significance of modern analytical techniques, international regulatory frameworks, and innovative traceability systems in addressing the current and future challenges in the coffee industry, aiming to enhance product quality, authenticity, and sustainability (Figure 7).

## 7. Kaldi Award 2023

The Kaldi Award, inspired by the legendary story of Kaldi, an Ethiopian goatherd, celebrates a seminal moment in coffee history, albeit one shrouded in myth and speculation. The widely-known Western narrative depicts Kaldi discovering coffee’s stimulating effects after observing his goats’ lively behavior upon eating berries from a bush [37]. Antonio Fausto Naironi, in his 1671 treatise “De saluberrima potione cahue, seu cafe nuncupate discursus”, popularized the tale in which Kaldi, intrigued by the berries’ impact, shared them with a monastery’s holy man. The narrative continues with the holy man discarding the berries into a fire, from which an alluring aroma arose, leading to the extraction of roasted beans and the brewing of the first coffee [37,38]. However, some skepticism surrounds this story, especially the monastery’s involvement. A more plausible assumption, aligning with the authors’ perspective, suggests that Kaldi himself may have serendipitously discovered coffee’s aromatic potential. This could have occurred through a simpler, more direct encounter, possibly involving the accidental roasting of beans in a fire used for daily needs, such as the burning of goat dung containing digested coffee cherries.

Despite its mythical aspects, the story of Kaldi remains a celebrated part of coffee culture. Reflecting this spirit, the Kaldi Award, initiated by Coffee Consulate and decided by an international jury of coffee experts, honors significant scientific contributions to the coffee industry. The award acknowledges the advancements and research that continue to shape our understanding and enjoyment of coffee, much like the legendary Kaldi’s initial encounter with this now-global phenomenon.

The Kaldi Award 2023 honors distinguished professionals for their exceptional contributions to the field of coffee science and the coffee industry.

Lifetime Achievement Award: Prof. Dr. Adriana Farah’s research on coffee ingredients and their nutritional and health effects has profoundly enhanced our understanding of coffee’s impact on human well-being. Her commitment and comprehensive studies in numerous, highly cited publications have significantly advanced global knowledge in the coffee field.

Green Coffee/Cultivation and Processing: Dr. Christophe Montagnon’s work on the genetics of coffee plants has been pivotal. His pioneering research has contributed to the discovery of more resilient and sustainable coffee varieties, addressing critical challenges in cultivation and processing.

Roasting and Analytics: Jan Teipel is recognized for his leadership in the development of standardized methods for coffee analyses, particularly the European Standard DIN EN 17992 [39], which uses Nuclear Magnetic Resonance (NMR) spectrometric techniques. His contributions have greatly improved the precision and reliability of coffee authenticity assessments, setting new benchmarks in the industry.

Preparation and Consumption: Dr. Amaia Iriondo-DeHond’s research on the importance, food safety, and applicability of coffee by-products has opened innovative pathways for their utilization. Her work has significantly contributed to sustainability in the coffee industry, showcasing the potential of coffee by-products beyond their conventional uses.

Special Award (Coffee Ambassador): The Kaldi Award 2023 also included a Special Award category, the Coffee Ambassador, which was granted for the first time. This honor was bestowed upon Dr. Massimiliano Fabian in recognition of his active role in shaping cooperation within global coffee organizations, including the International Coffee Organization (ICO). Dr. Fabian’s efforts have significantly contributed to fostering the collaborative networks and partnerships essential for the advancement of the coffee sector on an international level.

**Figure 8:**
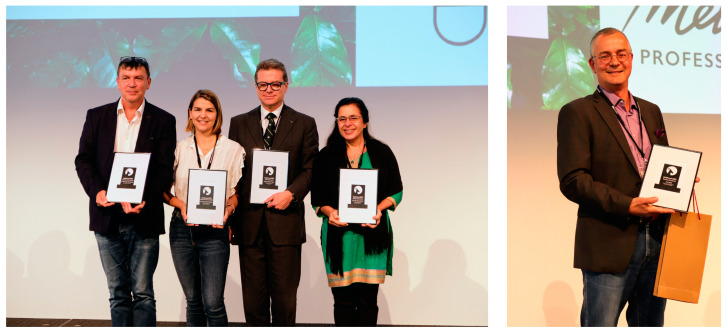
Kaldi Award laureates 2023 ((**left panel**), from left to right: Montagnon, Iriondo-deHond, Fabian, Farah; (**right panel**): Teipel). Permission has been obtained from all individuals in the image.

This year’s Kaldi Award recipients (Figure 8) have made influential contributions that extend beyond their specific research areas, impacting the coffee industry and shaping global practices. The accomplishments of these laureates exemplify the esteemed principles embodied by the Kaldi Award’s history, as detailed in Table 1, underscoring their commitment to excellence and innovation within the realms of coffee science and the broader industry.

## 8. Conclusions

The International Coffee Convention 2023 successfully illuminated the multifaceted nature of the global coffee industry, emphasizing the critical interplay between sustainability, health, technological innovation, and regulatory frameworks. The convention brought to light the increasing importance of *Coffea liberica* as a climate-resilient species, underscoring the need for biodiversity in coffee production as a response to climate change. The sessions highlighted the potential of coffee by-products to promote sustainability, revealing their untapped value in both health and environmental contexts.

Advancements in the coffee business and technology were showcased, reflecting the dynamic shifts in consumption patterns and the evolving landscape of coffee-related technologies. The discussions on coffee and health presented a nuanced view of coffee’s impact on human well-being, affirming its significant role in health promotion. The redefinition of these by-products, traditionally viewed as waste, into valuable resources reflects a paradigm shift, aligning with contemporary sustainability objectives.

The convention also delved into the complexities of coffee’s traceability and authentication, and the challenges posed by changing global regulations, especially in terms of deforestation policies and the traceability of coffee value chains. These discussions pointed to an urgent need for a greater alignment of practices and regulations to ensure a sustainable and transparent coffee market.

In conclusion, the International Coffee Convention 2023 successfully bridged the gap between various stakeholders in the coffee industry, fostering a deeper understanding of the challenges and opportunities that lie ahead. The truly interdisciplinary insights gained from this convention are invaluable in guiding future research, policy-making, and industry practices. Looking ahead, the coffee community eagerly anticipates the next International Coffee Convention in 2024 (17 October–18 October in Mannheim, Germany), where these critical discussions will continue, further shaping the future of coffee in a rapidly evolving world.

## Figures and Tables

**Table 1:** Previous and current Kaldi Award laureates.

Category	2016	2018	2020	2023
Lifetime Achievement	Richard von Hünersdorff (Great Britain): research and creation of the indispensable work “Coffee—a bibliography”	Ennio Cantergiani (Switzerland): research for the identification of roast aromas	Anita Vietri (Switzerland): the Anita Vietri Collection	Prof. Dr. Adriana Farah (Brazil): research on coffee ingredients and their nutritional and health effects
Green Coffee	Dr. Flávio Meira Borém (Brazil): development of modern protective packaging for green coffee	Dr. Kenny Lee Wee Ting (Malaysia): commitment to the research and preservation of Liberica coffee	Dr. Björn Schäfer (Germany): creation of the international protection collection of coffee varieties in the botanical garden of the Wilhelma in Stuttgart	Dr. Christophe Montagnon (France): work on the genetics in coffee plants
Roasting	Ram Evgi (Israel): thermodynamic roasting drum with perforations	Prof. Dr. Matthias Rädle (Germany): research on the identification of roast aromas by Raman spectroscopy	Rolf Kammerer (Germany): development of the Tyboon infrared roaster	Jan Teipel (Germany): development of a standardized method for the analysis of coffee by NMR
Preparation	Enrico Maltoni (Italy): the Enrico Maltoni Collection (Mumac)	Bjørn Møller (Denmark): development of an automatic coffee brewing machine for all filter and French press processes	Dr. Jörg Rieke-Zapp (Germany): development of the RS-16 intense extraction glass filter	Dr. Amaia Iriondo-DeHond (Spain): research on the importance and applicability of coffee by-products
Special Award (Coffee Ambassador)	-	-	-	Dr. Massimilano Fabian (Italy): the active shaping of cooperation between global coffee organizations

## Data Availability

No new data were created or analyzed in this study. Data sharing is not applicable to this article.
